# CRUSTED PILOLEIOMYOMA WITH MENTAL RETARDATION: A RARE ASSOCIATION

**DOI:** 10.4103/0019-5154.48995

**Published:** 2009

**Authors:** Sangeeta Kamboj, Raj Kumar Sharma, Amit Kumar, Shyam Sundar Chaudhary, Vir Kumar Jain

**Affiliations:** *From the Department of Dermatology, Rajendra Institute of Medical Sciences, Ranchi, India*; 1 *From the Department of General Surgery, Rajendra Institute of Medical Sciences, Ranchi, India*

**Keywords:** *Crusting*, *mental retardation*, *piloleiomyoma*

## Abstract

Piloleiomyoma is an uncommon benign smooth muscle neoplasm arising from arrector pili muscle. It is clinically defined by the presence of solitary or multiple reddish brown, dome-shaped, smooth papules or nodules, ranging in size from a few millimeters to a centimeter. The patients are otherwise healthy; but mental retardation developing in some patients with multiple Piloleiomyomas has been emerging as an intriguing matter for analysis by the scientists. In this case report, a mentally retarded patient with Piloleiomyoma is described, who, besides the characteristic smooth and dome-shaped lesions on the anterolateral aspect of the dorsum of the right foot, had developed crusting on one of the largest lesions. The histopathological features were consistent with Piloleiomyoma. The occurrence of Piloleiomyoma in a mentally retarded child and its unusual crusted nature has been rarely reported. The association between Piloleiomyoma and mental retardation is further stressed in this case report.

## Introduction

Piloleiomyoma as the name suggests arises from arrectores pilorum muscle, which is a smooth muscle component of the pilosebaceous unit. The primary lesion is multiple dome-shaped reddish brown nodules or a plaque with a smooth and shiny surface situated on the face, back or extremities. Secondary changes such as crusting or ulceration has been rarely seen. Multiple lesions may involve more than one part of the body. Sensitivity to cold or touch correlates well with the mechanism of goose flesh, brought about by the contraction of the arrector pili muscle.

## Case Report

**Figure 1:**
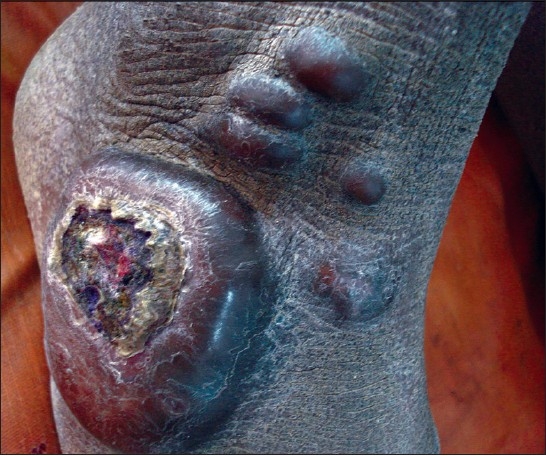
Multiple piloleiomyoma on the dorsum of the foot

A 17-year-old male born of non consanguineous marriage presented with multiple nodules on the dorsum of the right foot, for two years. There was history of spontaneous pain intermittently and also pain on exposure to cold and trauma. The patient was mentally subnormal since childhood, as compared to others of his age. On examination, there was a large swelling of 7cm × 5cm in size, present on the anterolateral aspect of the dorsum of the right foot. Four to five smaller nodules were present adjacent to it. The lesions were well defined, with a smooth surface and firm in consistency. The larger lesion showed central crusting which had occurred, due to spontaneous rupture, two months previously. Swelling was not fixed to the underlying structure [Figure 1]. No other skin or mucus membrane lesion was found. There was no family history of similar lesions. The patient was referred to the department of psychiatry for evaluation of mental intelligence. Applying the WHO criteria, he was diagnosed as having moderate mental retardation. The diagnosis was done after assessment using the Stanford Binet and Bhatia Battery of Intelligence tests, which showed his IQ (Intelligence Quotient) levels to be 38 and 44 respectively. His Magnetic Resonance Imaging (MRI) showed all the structures within normal limits.

**Figure 2:**
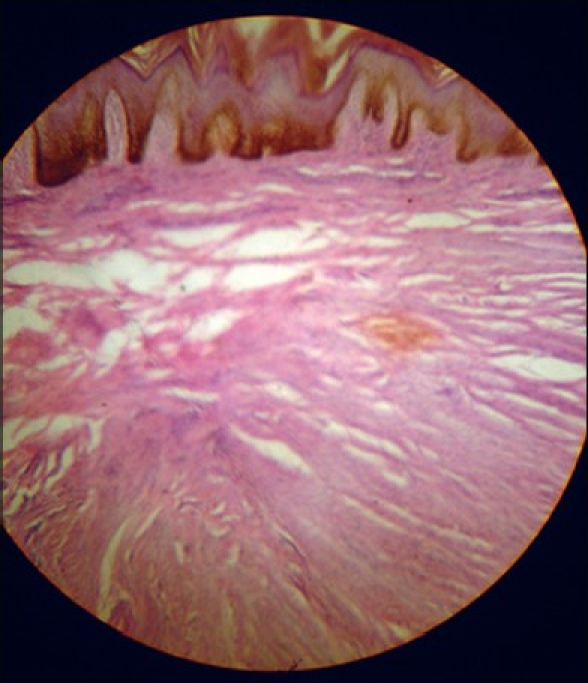
Histopathological picture (H & E ×40)

Routine investigations were within normal limits. Excision biopsy of the lesion revealed interlacing bundles of smooth muscle fibers, with variable amounts of collagen bundles in the dermis. Muscle fibers were straight, without any waviness with central, elongated nuclei, and, thus, a histopathological diagnosis of Piloleiomyoma was made [Figure 2].

The lesions were excised en masse, followed by split skin grafting. During surgery, the lesions were found above the deep fascia and were easily separated from it. Post operative recovery was uneventful.

## Discussion

Leiomyomas are benign soft tissue neoplasms that arise from smooth muscle and were first described by Virchow in 1854. The onset is in the second or third decade of life. They usually appear as reddish brown papulo-nodules. Patients may develop several hundred lesions. Piloleiomyomas are believed to arise from the arrector pili muscle of the pilosebaceous unit. The arrector pili muscle, from which Piloleiomyoma originates, attaches proximally to the hair follicle and distally to multiple attachment points within the papillary and reticular dermis, as well as to the basement membrane. Piloleiomyomas can plausibly emerge from each of these various points of insertion and occur as multiple tumors. These tumors are painful on exposure to cold and trauma and pain could result from local pressure by the tumor on cutaneous nerves. However, the histological findings do not show that prominent nerve fibers are associated with these tumors. Yet, some have suggested that muscle contraction may be pivotal in the induction of pain. The excitation of the arrector pili muscle occurs via the sympathetic nervous system which secretes Norepinephrine, by postganglionic nerve fibers, thus activating the alpha-receptors of the muscle. Subsequently, muscle contraction ensues, which is triggered by the influx of calcium ions. Understanding this basic physiologic process may be relevant to the medical treatment of symptomatic leiomyomas.

An association between multiple cutaneous leiomyomas and mental retardation was described by Fryns *et al*. in 1985, where they described chromosomal abnormality of 9p trisomy/18p monosomy as a probable genetic basis of this association.[1] A genetic underpinning of leiomyoma has long been foreseen and the notion has been strengthened by identification of genetic loci, with a strong linkage to it. The most compelling evidence for its genetic basis comes from a recent identification of the MCUL 1 locus on chromosome 1q 42.3-43 in 10 families that had multiple cutaneous leiomyomas and uterine fibroids,[2] and by the identification of two pedigrees with a specific combination of renal cell carcinoma and uterine and cutaneous leiomyomas linked to the same loci.

Our case brings up two issues to be discussed. First is the co-occurrence of mental retardation with Piloleiomyoma. Although mental retardation is often found with several neurocutaneous and tumor syndromes, its co-morbidity Piloleiomyoma has been reported only a couple of times. The latest report was of a huge Leiomyoma in a woman with Down's syndrome.[3] Our patient did not show any features suggestive of Down's or other syndromes. This brings up the next important issue of an independent genetic basis of this co-morbidity. In spite of the fact that chance factors can be easily stressed in view of such a few occurrences, a genetic basis cannot be ruled out, in view of the strong genetic associations reported with Leiomyoma cases in general and, specifically, in conditions of its co-morbidity with mental retardation.[1,3] The limitation of facilities for a genetic workout inhibited us from establishing a genetic association in our case, which is seriously warranted in such cases and which weigh in favor of this association. Central crusting in Piloleiomyoma itself is a rare occurrence which was reported earlier.[4]

## Conclusion

To conclude, it will be appropriate to state that any case with skin tumors should be thoroughly assessed for other tumors and neuropsychiatric abnormalities, and a genetic assessment should be carried out whenever possible, as it can lead to further unraveling of genetic underpinnings of these disorders.
